# Ring chromosomes uncovered by optical genome mapping: impact of telomeric-associated regions and reference genome selection on structural variant interpretation

**DOI:** 10.1007/s10577-026-09806-5

**Published:** 2026-07-20

**Authors:** Bruna Burssed, André R. C. P. Oliveira, Fernanda Teixeira Bellucco, Maria Isabel Melaragno

**Affiliations:** 1 Genetics Division, Department of Morphology and Genetics, Universidade Federal de São Paulo, São Paulo, Brazil; 2 Uniscience do Brasil, Uniscience Molecular Laboratory, São Paulo, Brazil

**Keywords:** Ring chromosome, Optical genome mapping, Reference genome, Structural variants, Telomeric-associated regions, Telomere-to-telomere assembly

## Abstract

Ring chromosomes (RCs) are rare structural variants usually formed by fusion of both chromosome arm extremities, frequently associated with terminal deletions. RCs are investigated through karyotype, fluorescence in situ hybridization, chromosomal microarray analysis, and, more recently, optical genome mapping (OGM), which enables genome-wide structural variant detection; however, its accuracy depends on alignment to a reference genome and proper filtering of SVs. Here, two patients with previously characterized ring chromosomes 3 and 18, each with a terminal deletion in only one chromosome arm (3p and 18q), were analyzed by OGM after aligning the data against two reference genomes (GRCh38/hg38 and T2T-CHM13) and compared with long-read sequencing (LRS) results stemming from a parallel study that included both patients. Patient 1's RC3 was misinterpreted as a translocation between chromosomes 3 and 19 when analyzed with GRCh38/hg38. Reanalysis with T2T-CHM13 correctly identified the RC3 and demonstrated retention of the full 3q arm, including telomeric-associated regions, which was confirmed by LRS. Patient 2’s ring chromosome 18 was identified with GRCh38/hg38 only after lowering the confidence score filter, whereas T2T-CHM13 detected the RC18 immediately without involvement of 18p telomeric-associated regions. LRS diverged from this analysis by showing the presence of these regions in the RC18 due to its nucleotide-level resolution. Only eight out of ~ 3,000 RCs have been reported in the literature as resolved using OGM, and here we add two more cases analyzed with different reference genomes and highlight the techniques’ advantages and limitations for RC characterization. Since the T2T-CHM13 analysis outperformed GRCh38/hg38 because it resolves previously inaccessible telomeric regions, we included an investigation into which other chromosomes could also benefit more from this approach.

**Supplementary Information:**

The online version contains supplementary material available at 10.1007/s10577-026-09806-5.

## Introduction

**Fig. 1:**
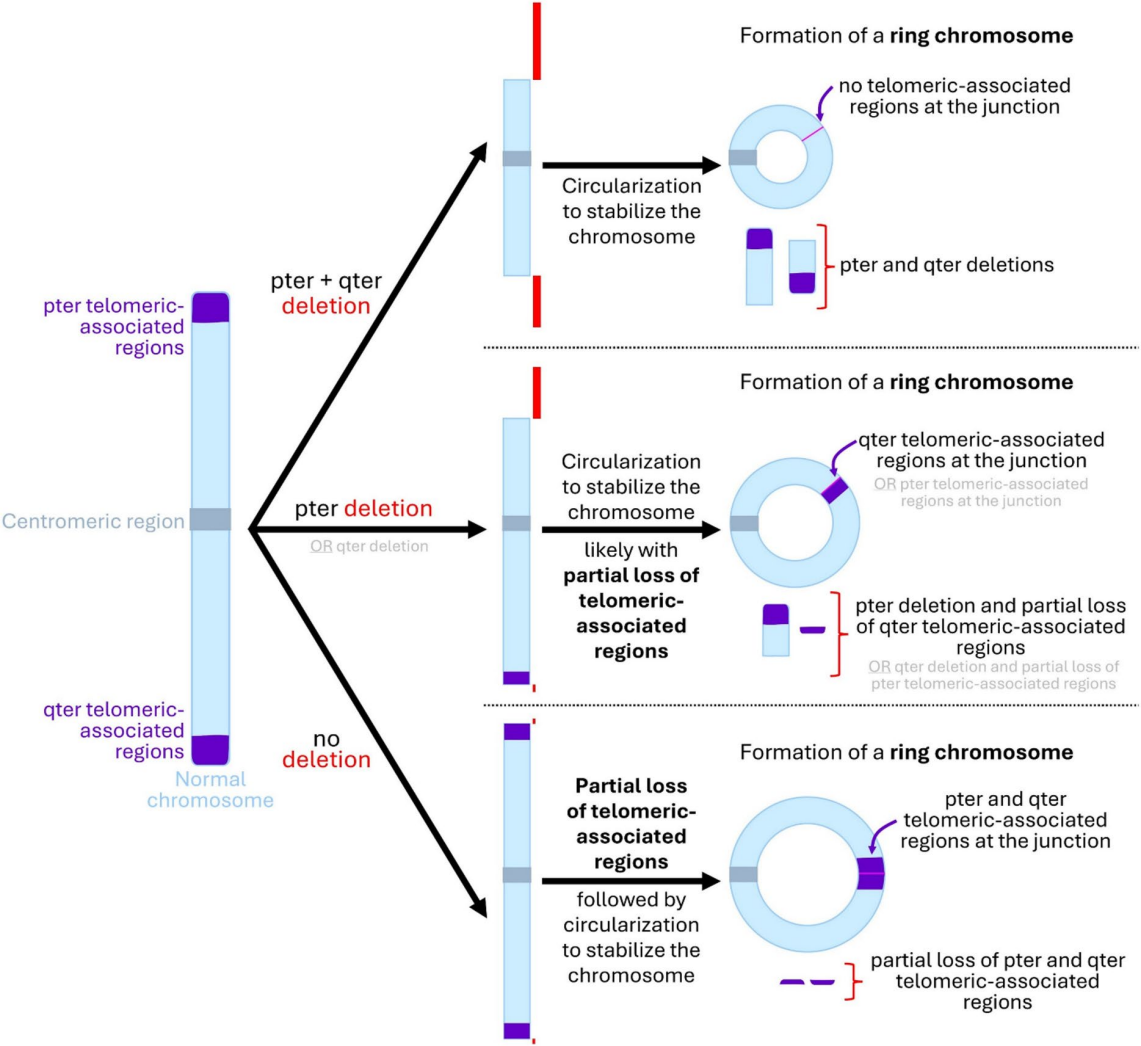
Mechanisms of ring chromosome formation and situation of telomeric-associated regions in the final rearranged chromosome. To the right, a normal chromosome with its telomeric-associated regions highlighted in purple (out of scale). Suffering terminal deletions in both chromosome arms (top panel), the chromosome may undergo circularization for its stabilization, forming a ring chromosome that presents no telomeric-associated regions at the junction point. When bearing a terminal deletion in only one chromosome arm (middle panel), the chromosome may suffer partial loss of the other arm’s telomeric-associated regions so it can also circularize for stabilization, forming a ring chromosome that presents telomeric-associated regions on one side of the junction point. Due to a partial loss of both arms’ telomeric-associated regions (bottom panel), a fusion may happen given the similarity in their repetitive sequences and circularize the chromosome for stabilization, forming a ring chromosome that presents telomeric-associated regions on both sides of the junction point. The first situation might be solved using the GRCh38/hg38 reference since it does not involve the complex telomeric regions. The second and third scenarios, however, may require the T2T-CHM13 reference since it presents the sequence of the telomeres and, therefore, can provide a more complete analysis of the involved regions

Ring chromosomes (RCs) are rare structural variants (SVs) formed by circular DNA molecules with an estimated incidence of 1 in 50,000 newborns (Li et al. 2022). RCs are a type of intrachromosomal rearrangement usually formed when the chromosome extremities fuse, either with or without terminal deletion at one or both ends (Guilherme et al. 2011a; Burssed et al. 2022). Chromosomes that undergo terminal deletions lose their telomere-associated regions and become vulnerable due to their genetic instability (Yu and Graf 2010; Burssed et al. 2022). To avoid this, the cells have procedures to repair and stabilize these chromosomes. The first two ways involve the chromosome regaining a telomere, either relying on telomerase to synthetize a new one or by capturing one from its sister chromatid or a non-homologous chromosome end, with the latter forming a translocation. The third way is by circularization, thus forming a ring chromosome (Yu and Graf 2010; Burssed et al. 2022) (Fig. 1). Alternatively, partial loss of the telomeric or subtelomeric regions of complete chromosomes may lead to their fusion due to their similar repetitive sequences and consequent formation of the circular DNA molecule. Both situations can also happen concomitantly with a terminal deletion at one end and partial loss of telomeric-associated regions in the other (Guilherme et al. 2011a; Burssed et al. 2022).

The phenotype of patients with RCs may vary based on the affected chromosome, the size of the deletion(s), and the secondary genetic alterations caused by ring chromosome instability (Guilherme et al. 2011a; Li et al. 2022). Despite this, RC patients can present ‘ring syndrome’, in which they share certain phenotypes, including growth failure, variable intellectual disability, and mild dysmorphism (Kosztolányi 1987, 2009). This condition likely happens due to the loss of cells with further genetic imbalances, such as the loss of a RC (McKinley et al. 1991; Kosztolányi 2009; Li et al. 2022).

Since the observation of the very first RC reported on the X chromosome in 1962 (Lindsten and Tillinger 1962), genetic and genomic technologies have seen numerous advancements that have enabled the identification and characterization of RCs (Li et al. 2022). Karyotype is the most commonly used methodology to detect RCs, with fluorescence in situ hybridization (FISH) and chromosomal microarray analysis (CMA) assisting in their characterization (Kim et al. 2025; Murry and DuPont 2025). However, higher resolution techniques, such as whole genome sequencing (WGS) and optical genome mapping (OGM), provide a more comprehensive analysis of the RC and may shorten the journey until the diagnosis (Kim et al. 2025; Murry and DuPont 2025).

Optical genome mapping is an emerging technique capable of detecting a myriad of chromosomal alterations, including aneuploidies, copy number variants (CNVs), and SVs either in a balanced or an unbalanced state (Dremsek et al. 2021; Mantere et al. 2021; Neveling et al. 2021). It relies on imaging ultra-long linearized DNA molecules labeled at specific motifs that are converted to maps that can be compared to a reference in order to detect the alteration (Mantere et al. 2021; Neveling et al. 2021).

The reference genome has been developing since the first human genome was sequenced in 2003 (Consortium 2004). Since then, the sequence has been updated due to breakthroughs in techniques to resolve gaps and provide more accuracy (Kaye and Wasserman 2021). The most frequently used reference genomes have been GRCh37/hg19 and GRCh38/hg38, released by the Genome Reference Consortium (GRC), and, more recently, the T2T-CHM13 reference, launched by the Telomere-to-Telomere (T2T) Consortium (Taylor et al. 2024). Each new release aims to refine the previous one so it can be used as a better standard against which results can be reported in a way that facilitates universal knowledge exchange between scientists (Kaye and Wasserman 2021; Taylor et al. 2024).

Here, we report two ring chromosome patients investigated by optical genome mapping that presented different genetic results depending on the filter and/or the reference genome used for the analysis due to the involvement of telomeric-associated regions.

## Methods

### Enrollment

Two patients with ring chromosomes 3 and 18 who were previously investigated with classical cytogenetics techniques were included in the study (Guilherme et al. 2011b, a). The samples used in this study were collected after written informed consent and approval of the local ethics committee (CAAE 40846114.2.0000.5505, CEP 0028/2015).

### Optical genome mapping (OGM)

Peripheral blood was collected into two EDTA tubes. Ultra-high molecular weight (UHMW) DNA was isolated from 650 μL of whole peripheral blood using the SP Blood and Cell Culture DNA Isolation Kit according to the manufacturer’s protocol (Bionano Genomics, San Diego, CA, USA). UHMW genomic DNA (gDNA) molecules were labeled with the DLS (Direct Label and Stain) DNA Labeling Kit (Bionano Genomics). After that, 750 ng of gDNA were labeled with Direct Label Enzyme (DLE-1) and DL-green fluorophores. After a wash-out of the DL-green fluorophores excess, the DNA backbone was counterstained overnight before quantitation. Labeled UHMW gDNA was loaded on a Saphyr chip for linearization and imaging on the Saphyr instrument (Bionano Genomics). The de novo assembly and variant annotation pipeline was executed with Bionano Solve software v.3.7. Reporting and direct visualization of structural variants were performed using Bionano Access software v.1.7. OGM was analyzed after alignment with reference genomes GRCh38/hg38 and T2T-CHM13. The UCSC Genome Browser (Casper et al. 2026) was used to analyze the regions where the breakpoints were located. In the GRCh38/hg38 assembly, we used tracks “Assembly from Fragments” and “Gap Locations”, while in the T2T-CHM13 assembly, we employed tracks “CHM13 unique in comparison to GRCh38/hg38” and “LiftOver alignments from CHM13 to hg38”. Technical performance of OGM for each patient can be found in Supplementary Table 1.

### Long-read sequencing (LRS)

Long-read sequencing for both patients was performed as part of separate research with the International Consortium for Human Ring Chromosomes (ICHRC) (Chong et al. 2025). Briefly, library-prepped reads of 15 to 20 kb added to Revio 24 M SMRT cells (PacBio, Menlo Park, CA, USA) were sequenced at the Yale Center for Genome Analysis (YCGA) on a PacBio Revio System (PacBio). Alignment to T2T-CHM13 was performed with pbmm2 (SMRT link v12.0.0). The algorithms pbsv and HiFiCNV were used for SV and CNV calling, respectively. Breakpoint location was performed based on these algorithms’ calls as well as visual inspection on the Integrative Genomics Viewer (IGV) 2.4.14 software (Broad Institute and the Regents of the University of California).

### Size analysis of terminal unlabeled regions

Since the detection of terminal deletions in ring chromosomes plays a significant role in their characterization by optical genome mapping, we analyzed the genome maps of all chromosomes to check the size of the unlabeled region between the chromosome extremities and their closest OGM label. All coordinates were annotated based on reference genomes GRCh38/hg38 and T2T-CHM13 on the Bionano Access software v.1.7. Coordinates corresponding to the chromosome extremities were considered as 0 (zero) for all pter regions and as the coordinate of the last base for each chromosome’s qter region, which matched to the ones reported by the National Center for Biotechnology Information (NCBI) (Goldfarb et al. 2025). Coordinates corresponding to the labeled regions were considered as the coordinate of the most upstream label and the most downstream label shown on the reference map of each chromosome for the pter and qter region, respectively. Sizes of unlabeled regions were obtained by subtracting the associated coordinates of each chromosome’s terminal regions.

## Results

### Patient 1

**Fig. 2:**
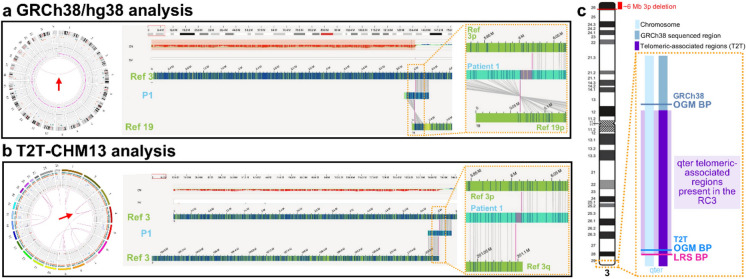
Results of patient 1. Images of circos plots show all 24 chromosomes with their idiograms or assigned color on the periphery. Images of the maps show reference maps in green and patient maps in blue. Annotations above may include idiograms and CNV/SV call tracks. In both images, pink lines represent fusions, either intra-chromosomal or inter-chromosomal. (**a**) Analysis using reference genome GRCh38/hg38. To the left, the OGM circos plot shows the detection of a translocation between chromosome 3 and chromosome 19 (red arrow). To the right, the OGM maps with a zoom highlighting the t(3;19) junction point. (**b**) Analysis using reference genome T2T-CHM13. To the left, the OGM circos plot shows the detection of a ring chromosome 3 (red arrow). To the right, the OGM maps with a zoom highlighting the RC3 junction point between 3p and 3q. The Bionano Access software did not have filters for the T2T-CHM13 reference at the time of the analysis, so the circos plot shows all alterations. (**c**) Schematic representation of the findings in chromosome 3. To the right, idiogram of chromosome 3 with the 3p deletion represented in red. The box on the right represents a zoom into the 3qter region (out of scale), showing the chromosome region sequenced in the GRCh38/hg38 reference (darker blue), flagging where the OGM breakpoint from this analysis was located, and the 3qter telomeric-associated regions sequenced on the T2T-CHM13 reference (purple), stressing the proximity between the OGM and LRS breakpoints detected in this reference. A lighter purple box identifies the 3qter telomeric-associated regions present in the ring chromosome 3

Patient 1 is a 33-year-old male who presents a ring chromosome 3 with a ~ 6 Mb deletion in the short arm (Guilherme et al. 2011b; Melaragno and Burssed 2024). OGM using reference genome GRCh38/hg38 revealed the same deletion but, surprisingly, the downstream breakpoint of the deletion joined with a region of chromosome 19p, thus indicating a translocation between chromosomes 3 and 19: t(3;19)(p26.1;p13.3) (Fig. 2a). The supposedly involved region of 19p is at the beginning of the chromosome (chr19:64,162) immediately downstream of the telomere (with ~ 60 kb in size in GRCh38/hg38).

This result was unexpected because karyotyping showed a ring chromosome, which should have been detected by OGM. Analyzing the 3qter region in the Bionano Access software, we notice that the last existing OGM label is far from the qter end of the reference map. This distance likely reflects the presence of a ~ 60 kb unsequenced gap/telomeric region annotated at the distal end of chromosome 3 in the UCSC Genome Browser, which would not generate labels in the GRCh38/hg38 analysis. The patient’s last aligned label at 3q maps in a region that was completely sequenced in the GRCh38/hg38 reference before the gaps/telomere, which may explain the numerous unaligned labels seen in his map.

Upon a reanalysis using T2T-CHM13 as reference, OGM detected the ring chromosome 3 with a 3p deletion: ogm[T2T] r(3)(p26.1q29)(6,012,432_201,097,227) (Fig. 2b). No involvement of chromosome 19 was found. This analysis confirmed that the 3q arm is fully present in the RC3 alongside its telomere-associated regions, and that is what prevented GRCh38-based analysis from detecting this ring chromosome (Fig. 2c).

Patient 1’s LRS analysis corroborates the T2T-CHM13 OGM findings, also showing the presence of 3q telomere-associated regions in the RC3. Comparing the location of the breakpoints detected by each technique, we identified a difference of 2,136 bp between the 3p breakpoints and of 6,596 bp between the 3q breakpoints (Table 1).

**Table 1:** Breakpoints detected for each patient based on technique and reference genome and difference between them

P	Confidence score of intra-fusion (ring detection)		Chr ends	GRCh38/hg38 breakpoints	T2T-CHM13 breakpoints		Difference between breakpoints (bp)	
	GRCh38/hg38	T2T-CHM13		OGM	OGM	LRS	hg38 OGM vs T2T OGM	T2T OGM vs T2T LRS
1	0.07 for t(3;19)	0.73	3p	chr3:6,018,675	chr3:6,012,432	chr3:6,010,296	2,243	2,136
			3q	chr19:64,162	chr3:201,097,227	chr3:201,103,823	-	6,596
2	0.00	0.30	18p	chr18:18,868	chr18:172,612	-^a^	153,744	-^a^
			18q	chr18:61,642,239	chr18:61,845,252	chr18:61,845,470	203,013	218

*P* patient, *Chr* chromosome, *OGM* optical genome mapping, *LRS* long-read sequencing, *bp* base pair; ^a^The 18p LRS breakpoint considered by Chong et al (2025) is chr18:55,654; however, since the chimeric reads align to multiple regions of 18pter, it is possible to make many breakpoint inferences, some closer to and some further from the OGM 18p breakpoint; therefore, no specific breakpoint is considered here

### Patient 2

**Fig. 3:**
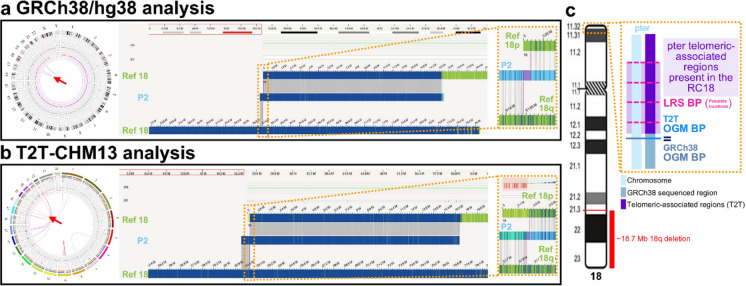
Results of patient 2. Images of circos plots show all 24 chromosomes with their idiograms or assigned color on the periphery. Images of the maps show reference maps in green and patient maps in blue. Annotations above may include idiograms and CNV/SV call tracks. In both images, pink lines represent fusions, either intra-chromosomal or inter-chromosomal. (**a**) Analysis using reference genome GRCh38/hg38. To the left, the OGM circos plot shows the detection of a fusion between regions 18p and 18q, indicating the ring chromosome, when the SV Confidence filter is set to “All” (red arrow). To the right, the OGM maps with a zoom highlighting the fusion junction point. (**b**) Analysis using reference genome T2T-CHM13. To the left, the OGM circos plot shows the detection of a ring chromosome 18 (red arrow). To the right, the OGM maps with a zoom highlighting the RC18 junction point between 18p and 18q. The Bionano Access software did not have filters for the T2T-CHM13 reference at the time of the analysis, so the circos plot shows all alterations. (**c**) Schematic representation of the findings in chromosome18. To the right, idiogram of chromosome 18 with the 18q deletion represented in red. The box on the right represents a zoom into the 18pter region (out of scale), showing the chromosome region sequenced in the GRCh38/hg38 reference (darker blue), emphasizing the same location where the OGM breakpoints from both references were located, and the 18pter telomeric-associated regions sequenced on the T2T-CHM13 reference (purple), where the LRS breakpoint is located. A lighter purple box identifies the 18pter telomeric-associated regions present in the ring chromosome 18 revealed only by LRS

Patient 2 is a 22-year-old female who presented a ring chromosome 18 with a ~ 18.7 Mb deletion in the long arm (Guilherme et al. 2011a). OGM using reference genome GRCh38/hg38 revealed only the 18q deletion and showed no indication of a ring chromosome when using the “Recommended” SV Confidence filter for intra-fusion in the Bionano Access software, with a closer inspection uncovering a patient map that shows an 18p-18q intrachromosomal fusion, thus suggesting a RC. Changing the SV Confidence filter to “All”, the RC18 with a del(18q) was then revealed in the GRCh38/hg38 analysis as r(18)(p11.32q21.33)(18,868_61,642,239) (Fig. 3a). Annotations in the 18pter region in the UCSC Genome Browser show that the unsequenced regions composed of gaps/telomeres present ~ 10 kb in size, therefore, the 18p RC breakpoint mapped to a region that was completely sequenced in the GRCh38/hg38 reference.

Reanalysis with the T2T-CHM13 reference revealed the ring chromosome 18 with 18q deletion: ogm[T2T] r(18)(p11.32q21.33)(172,612_61,845,252) (Fig. 3b). The 18p breakpoint is located in a region already sequenced in GRCh38/hg38; therefore, the OGM analysis led us to conclude that there are no telomeric-associated regions present in the RC18, which is also suggested by a lower copy number in the 18pter region seen in the T2T-CHM13 analysis.

Patient 2’s LRS analysis, on the other hand, revealed the presence of 18p telomeric-associated regions in the RC18 (Chong et al. 2025), diverging from the T2T-CHM13 OGM findings (Fig. 3c). The LRS 18p breakpoint is located in the telomere variant repeat (TVR) composed of (TAACCC)n. Although inferred that the 18q breakpoint joined the TVR at ~ 55.6 kb from the start of chromosome 18, the chimeric reads align to multiple regions of 18pter, leading to many possible breakpoint inferences, some closer to and some further from the OGM breakpoint. The LRS 18q breakpoint has a difference of only 218 bp from the OGM breakpoint (Table 1).

### Size analysis of terminal unlabeled regions

Considering the difference in the size of the telomeres and gaps at the terminal regions between each chromosome arm, we checked the sizes of the unlabeled region between the chromosome extremities and their closest OGM label in order to infer which chromosomes could benefit more from the T2T-CHM13 analysis when these regions are involved in rearrangements (Supplementary Table 2). Chromosomes 6 and 8 presented large unlabeled regions in both chromosome extremities and, therefore, would greatly benefit from the T2T-CHM13 analysis. Large unlabeled regions only on the p arm were seen in chromosomes 11, 17, and 19, while on only the q arm were present in chromosomes 1, 3, 5, 9, 12, 16, and 18. The chromosomes whose unlabeled regions were small in both arms are 2, 4, 7, 10, and X, therefore, we infer that analysis using the GRCh38/hg38 could possibly solve rearrangements involving them. The acrocentric chromosomes’ short arms still present a challenge in their alignment even on the T2T-CHM13 analysis. These regions are better mapped using this reference, but, for both OGM and LRS, it is sometimes difficult to assign a sequence to a single acrocentric short arm, requiring a careful analysis and interpretation of the data.

## Discussion

The detection of variations usually requires the comparison of a query to a reference. In the field of genetics, the reference genome is a key factor in nearly all clinical, comparative, and population genomic analyses (Taylor et al. 2024). Having a reference sequence against which we align sequencing reads, or optical maps, facilitates the process of detecting variants based on alignment mismatches, particularly when compared to performing a de novo assembly of a genome sequence (Taylor et al. 2024). In addition, the reference genome creates a shared coordinate system so the comparison with an individual’s genome sequence can be standardized across all genomic analyses (Taylor et al. 2024).

In 2003, the genome sequence (build 35) released by the Human Genome Project (HGP) contained 2.85 billion nucleotides and covered 99% of the euchromatic genome (Consortium 2004; Taylor et al. 2024). The Genome Reference Consortium (GRC) released two important genome assemblies: the GRCh37/hg19 (Genome Reference Consortium Human Build 37) reference genome, which was launched in 2009 and contained 3.13 billion nucleotides, with a gap length of ~ 239 Mb, and the GRCh38/hg38 (Genome Reference Consortium Human Build 38), which was released in 2013 containing 3.2 billion nucleotides and ~ 159 Mb of unresolved sequences. (Schneider et al. 2017; Taylor et al. 2024). Despite being longer and more contiguous than the previous assemblies, the GRCh38/hg38 reference still presents limitations, as the large stretches of unresolved sequences are represented by manufactured sequences with features that mimic their true repeat content and it contains several errors, mainly involving the genome’s complex regions, such as segmental duplications, centromeres, and telomeres, offering more challenges in the analysis when these are involved in the rearrangements (Taylor et al. 2024). The Telomere-to-Telomere (T2T) Consortium was established in 2019 to improve the reference genome by providing a more complete and accurate assembly (Taylor et al. 2024). In 2022, they released the T2T-CHM13 reference, which addresses the remaining 8% of the genome, mainly composed of heterochromatic regions, such as the centromeric and telomeric ones, the short arms of acrocentric chromosomes, and other complex and repetitive sequences (Nurk et al. 2022; Taylor et al. 2024). The complete Y chromosome was available a year later (Rhie et al. 2023). The T2T-CHM13 reference presents no gaps and is composed of 3.055 billion nucleotides (Nurk et al. 2022).

Each new assembly of the reference genome adds information and accuracy for the detection of variation (Taylor et al. 2024). The inclusion of complex regions, such as the telomeres and the acrocentric chromosomes’ short arms, in the T2T-CHM13 reference allowed for better characterization of genomic alterations involving these regions.

The detection of ring chromosomes has benefitted from the employment of the T2T-CHM13 reference when applying optical genome mapping, which is able to uncover the terminal deletion(s) with the CNV pipeline, based on normalized molecule coverage, while the junction point to form the RC is determined as a fusion with the SV pipeline, which compares the labeling patterns and distances between the sample’s map with the reference (Mantere et al. 2021). The choice of reference genome for the OGM analysis is key since the more complete the reference, the more matched labels we can achieve. Since the involvement of telomeric-associated regions in the RC poses a challenge for characterization given the complexity of these regions, the T2T-CHM13 reference emerged as an asset to solving this problem.

Both patients described here presented RCs, one in chromosome 3 and the other in chromosome 18, with a terminal deletion in only one chromosome arm detected by CMA (3q and 18q, respectively). Therefore, the telomeric region of the opposite arm appeared to be present in the final rearrangement.

This interpretation was confirmed for Patient 1 after the T2T analysis by OGM. Using GRCh38, the patient’s map had its labels aligned to chromosome 3p and 19p; therefore, a t(3;19) was called. Nevertheless, the patient’s map still presented unmatched labels to both regions, reiterating that the result should not be fully trusted. The T2T-CHM13 reference allowed for proper RC detection and confirmation of breakpoint locations since Patient 1’s 3q breakpoint was found in a region sequenced only by this reference, corresponding to a telomeric-associated region, which was not present in the GRCh38 assembly. Consequently, we can conclude that the RC3 retains telomeric-associated sequences, which was also confirmed with LRS (Fig. 2c).

For Patient 2, the interpretation was invalid based on the OGM investigation, which shows indication of a RC18 in all analyses because both RC breakpoints were detected in already sequenced regions. The T2T analysis corroborated that Patient 2’s 18p breakpoint was in a region that was sequenced in GRCh38/hg38 and whose upstream region seemed to be deleted, thus indicating no involvement of telomeric-associated regions in the RC. The LRS analysis, on the other hand, showed a different result for the 18p region. The 18p breakpoint found through LRS is located in the TVR (TAACCC)n with multiple coordinates being suggested as the breakpoint. Chong et al (2025) highlighted this and inferred the breakpoint at chr18:55,654, which would lead to a ~ 117 kb distance from the OGM breakpoint. However, the uncertainty region in the patient’s OGM map present a size of ~ 19.2 kb, indicating that the actual breakpoint should not be that far from the OGM-located one. In addition, based on the UCSC Genome Browser annotations, we observed that the 18p terminal region is full of segmental duplications that match amongst themselves in this region, hampering read alignments and breakpoint detection. Despite not being able to precisely define the 18p breakpoint, evidence shows that it is located in a TVR, implying that the RC18 also retains telomeric-associated sequences.

Patient 2’s OGM analysis using GRCh38/hg38 also shows the importance of checking the filters used in the Bionano Access software, since changing the SV Confidence from “Recommended” to “All” resulted in the software being able to call the RC18, whose intrachromosomal fusion has a confidence score of zero. The “Recommended” option filters out SVs with low confidence scores, such as the ones in or close to structurally complex regions, a factor that affects the SV confidence (Bionano Genomics 2021). It is important to be careful with lowering the filters, though, since false positive annotations may appear, mainly due to repetitive regions in the genome. Alignment to the T2T-CHM13 reference likely increases variant calling confidence scores (Banu et al. 2025), which then improves SV detection. In this case, the SV confidence score of the intrachromosomal fusion of the RC18 increased from zero in GRCh38 to 0.3 in T2T (Table 1).

The Ring Chromosome Database compiled information of over 3,000 RCs reported in the literature to date (Liehr and Li 2025; Liehr 2026). Among these, nine have been analyzed with OGM, with eight being fully resolved with the technique (Mantere et al. 2021; Schuy et al. 2024; Mostovoy et al. 2024; Kim et al. 2025) (Supplementary Table 3). In an investigation of the ability of OGM to detect known constitutional chromosomal alterations, Mantere et al. (2021) included in the cohort a mosaic RCX, which OGM was able to identify. Schuy et al. (2024) reported a patient with RC21 and terminal 21q deletion that was resolved with the association of OGM and long-read sequencing using the T2T-CHM13 reference genome. Mostovoy et al. (2024) also relied on this reference genome to solve five RCs with OGM, with a RC21 not being fully characterized. Kim et al. (2025) reported a RC17 with a 17p deletion through OGM. Interestingly, the RC was detected using the GRCh37/hg19 reference despite presenting a complete 17q arm with telomeric region. This was possible because chromosome 17 appears to present a more extensive coverage of the telomeric region, with the final label at 17q sitting only 1,049 nucleotides from the final reported nucleotide (Dyer et al. 2025), as similarly reported for GRCh38/hg38 in Supplementary Table 2, which shows a large unlabeled region only for the 17pter region.

Our data show that OGM can be used to analyze RCs since most breakpoints found by the technique were not far from the actual breakpoints found by LRS. The technique appeared as a solution that could replace karyotyping, chromosomal microarray analysis, and multiple rounds of FISH (Dremsek et al. 2021; Levy et al. 2025). However, the OGM detection of a ring chromosome can sometimes be based on inference when it detects an intra-fusion; therefore, karyotyping remains an important piece and ally in RC detection. For the present study’s patients, had we not performed karyotype, we might have trusted the initial GRCh38/hg38 analysis and informed the patients’ results as a t(3;19) and a del(18q) since these SVs presented high confidence in the OGM analysis. Because karyotyping was employed and a ring chromosome was detected, we knew these initial rearrangements were incorrect or missing information and continued our investigation using T2T-CHM13. At the time of the analysis, the Bionano Access software did not present the options of filtering for the T2T assembly, so the circos plot presents multiple alterations that could be false or common in the general population (Fig. 2b, 3b). Therefore, only using the T2T-CHM13 reference without karyotype analysis would also result in inaccuracy of the investigation. We believe that the cytogenetic analysis was essential for guiding the OGM analysis and resolution of both RC cases.

It’s important to note that the OGM technique is not without its intrinsic limitations. Since it requires ultra-high molecular weight DNA, it possesses a specific method of DNA extraction that is not commonly used in laboratories; therefore, most stored DNA is not suitable for OGM (Levy-Sakin et al. 2019; Kernohan and Boycott 2024). Both patients in this study had their DNA stored in our laboratory for years and had to be recalled to draw blood so they could be studied with OGM. There is also a shortness of control individuals to compare the detected SVs (Kernohan and Boycott 2024), and the most noteworthy limitation of the technique appears when it is compared with sequencing technologies, especially long-read sequencing. Since OGM is not a methodology based on sequencing but on labelling and imaging, it is not possible to detect single nucleotide variants or investigate the SVs’ breakpoints at the nucleotide level, as it only provides a range of genomic coordinates where the breakpoint may be (Kernohan and Boycott 2024; Levy et al. 2025). Interestingly, OGM is known for having the potential to detect low-level mosaicism, with reports showing the detection of fractions of 25–27% (Brakta et al. 2023; Orellana et al. 2025), though not when it presents fractions lower than 5% (Blancke et al. 2026). Ring chromosomes may present instability during meiosis that leads to dynamic mosaicism, showing different fractions above 5% but containing multiple different alterations between metaphasis (Kosztolányi 1987; Guilherme et al. 2011a), which could hamper the OGM investigation. Despite the limitations, OGM has an advantage in terms of cost (Levy et al. 2025), which is a main factor when deciding which methodology to apply in a study, especially in conditions where funding is limited. Our findings suggest, therefore, that the OGM analysis of ring chromosomes’ structure and CNVs is possible, given the eight previously solved cases and the two described here, and could be implemented replacing CMA and FISH for these cases while still remaining associated with karyotyping for improved detection and confirmation.

In this work, we enhanced the investigation of two ring chromosomes with the use of optical genome mapping and highlighted the importance of choosing the most appropriate reference genome for the analysis, especially when the SV involves telomeres or other repetitive regions. The T2T-CHM13 reference genome was crucial for the RC3 detection, revealing the involvement of telomeric-associated regions, while both GRCh38/hg38 and T2T-CHM13 were able to detect the RC18, though diverging from the ultimate LRS result. The cytogenetic analysis was also critical for the accurate analysis of the RCs. Few RC patients have been reported in the literature as investigated by OGM, and our study aims to include more cases in this group analyzed with different reference genomes, highlight the techniques’ advantages and limitations for RC characterization.

## Supplementary Information

Below is the link to the electronic supplementary material.

Supplementary file1 (DOCX 36 KB)

## Data Availability

The data that support the findings of this study are available from the corresponding author upon reasonable request.

## Abbreviations

*CMA*: Chromosomal microarray analysis
*CNVs*: Copy number variants
*DLS*: Direct label and stain
*FISH*: Fluorescence in situ hybridization
*gDNA*: Genomic DNA
*GRC*: Genome Reference Consortium
*GRCh37/hg19*: Genome Reference Consortium Human Build 37
*GRCh38/hg38*: Genome Reference Consortium Human Build 38
*HGP*: Human Genome Project
*ICHRC*: International Consortium for Human Ring Chromosomes
*LRS*: Long-read sequencing
*NCBI*: National Center for Biotechnology Information
*OGM*: Optical genome mapping
*RCs*: Ring chromosomes
*SVs*: Structural variants
*TVR*: Telomere variant repeat
*T2T*: Telomere-to-telomere
*T2T-CHM13*: Telomere-to-telomere complete hydatidiform mole 13
*UHMW*: Ultra-high molecular weight
*WGS*: Whole genome sequencing
