# Supplementary material for: Ring chromosomes uncovered by optical genome mapping: impact of telomeric-associated regions and reference genome selection on structural variant interpretation
Source: Chromosome Res. 2026 Jul 20;34(1):15. doi: 10.1007/s10577-026-09806-5 (PMC13384989; doi:10.1007/s10577-026-09806-5)
Supplement: Supplementary file 1 — Supplementary file1 (DOCX 36 KB) [file 10577_2026_9806_MOESM1_ESM.docx]

**Supplementary Table 1. Technical performance of optical genome mapping for each patient.**

| **Patient** | **Reference Genome** | **Molecule N50** | **Effective coverage of reference (×)** | **Label density (/100 kb)** | **Fraction of molecules aligned to assembly (Map Rate %)** |
| --- | --- | --- | --- | --- | --- |
| 1 | GRCh38/hg38 | 354.40 kb | 69.25 | 15.59 | 89.60 |
|  | T2T-CHM13 | 354.07 kb | 88.75 | 16.35 | 89.90 |
| 2 | GRCh38/hg38 | 303.04 kb | 82.75 | 16.98 | 76.80 |
|  | T2T-CHM13 | 303.04 kb | 76.83 | 17.24 | 81.90 |

**Supplementary Table 2. Coordinates of chromosome extremities and of their closest optical genome mapping label from Bionano Access and differences between them for all 24 human chromosomes.**

| **Chr** | **Region** | **GRCh38/hg38 coordinates** | | | **T2T-CHM13 coordinates** | | | **Increase in labeled regions from GRCh38/hg38 to T2T-CHM13 (bp)** |
| --- | --- | --- | --- | --- | --- | --- | --- | --- |
|  |  | **Chr extremity** | **Closest label** | **Size of unlabeled region (bp)** | **Chr extremity** | **Closest label** | **Size of unlabeled region (bp)** |  |
| **1** | **pter** | 0 | 14,453 | 14,453 | 0 | 4,561 | 4,561 | 9,892 |
|  | **qter** | 248,956,422 | 248,908,524 | 47,898 | 248,387,328 | 248,381,861 | 5,467 | 42,431 |
| **2** | **pter** | 0 | 15,924 | 15,924 | 0 | 9,297 | 9,297 | 6,627 |
|  | **qter** | 242,193,529 | 242,181,357 | 12,172 | 242,696,752 | 242,692,101 | 4,651 | 7,521 |
| **3** | **pter** | 0 | 12,290 | 12,290 | 0 | 4,559 | 4,559 | 7,731 |
|  | **qter** | 198,295,559 | 198,230,596 | 64,963 | 201,105,948 | 201,097,227 | 8,721 | 56,242 |
| **4** | **pter** | 0 | 14,701 | 14,701 | 0 | 6,117 | 6,117 | 8,584 |
|  | **qter** | 190,214,555 | 190,202,564 | 11,991 | 193,574,945 | 193,570,257 | 4,688 | 7,303 |
| **5** | **pter** | 0 | 19,314 | 19,314 | 0 | 9,782 | 9,782 | 9,532 |
|  | **qter** | 181,538,259 | 181,460,943 | 77,316 | 182,045,439 | 182,041,621 | 3,818 | 73,498 |
| **6** | **pter** | 0 | 76,216 | 76,216 | 0 | 4,439 | 4,439 | 71,777 |
|  | **qter** | 170,805,979 | 170,739,897 | 66,082 | 172,126,628 | 172,120,918 | 5,710 | 60,372 |
| **7** | **pter** | 0 | 10,487 | 10,487 | 0 | 5,673 | 5,673 | 4,814 |
|  | **qter** | 159,345,973 | 159,334,984 | 10,989 | 160,567,428 | 160,563,484 | 3,944 | 7,045 |
| **8** | **pter** | 0 | 61,805 | 61,805 | 0 | 4,662 | 4,662 | 57,143 |
|  | **qter** | 145,138,636 | 145,076,125 | 62,511 | 146,259,331 | 146,254,126 | 5,205 | 57,306 |
| **9** | **pter** | 0 | 14,566 | 14,566 | 0 | 8,367 | 8,367 | 6,199 |
|  | **qter** | 138,394,717 | 138,334,464 | 60,253 | 150,617,247 | 150,612,152 | 5,095 | 55,158 |
| **10** | **pter** | 0 | 18,514 | 18,514 | 0 | 11,522 | 11,522 | 6,992 |
|  | **qter** | 133,797,422 | 133,785,266 | 12,156 | 134,758,134 | 134,752,905 | 5,229 | 6,927 |
| **11** | **pter** | 0 | 60,457 | 60,457 | 0 | 6,015 | 6,015 | 54,442 |
|  | **qter** | 135,086,622 | 135,069,545 | 17,077 | 135,127,769 | 135,117,687 | 10,082 | 6,995 |
| **12** | **pter** | 0 | 14,568 | 14,568 | 0 | 7,569 | 7,569 | 6,999 |
|  | **qter** | 133,275,309 | 133,246,105 | 29,204 | 133,324,548 | 133,321,298 | 3,250 | 25,954 |
| **13** | **pter** | 0 | 18,174,796 | 18,174,796^a^ | 0 | 4,996,501 | 4,996,501^b^ | 13,178,295 |
|  | **qter** | 114,364,328 | 114,352,102 | 12,226 | 113,566,686 | 113,561,140 | 5,546 | 6,680 |
| **14** | **pter** | 0 | 16,004,725 | 16,004,725 ^a^ | 0 | 2,589 | 2,589 ^b^ | 16,002,136 |
|  | **qter** | 107,043,718 | 106,873,282 | 170,436 | 101,161,492 | 101,148,575 | 12,917 | 157,519 |
| **15** | **pter** | 0 | 19,801,760 | 19,801,760 ^a^ | 0 | 6,170 | 6,170 ^b^ | 19,795,590 |
|  | **qter** | 101,991,189 | 101,976,509 | 14,680 | 99,753,195 | 99,745,805 | 7,390 | 7,290 |
| **16** | **pter** | 0 | 14,134 | 14,134 | 0 | 7,060 | 7,060 | 7,074 |
|  | **qter** | 90,338,345 | 90,224,751 | 113,594 | 96,330,374 | 96,325,431 | 4,943 | 108,651 |
| **17** | **pter** | 0 | 66,653 | 66,653 | 0 | 2,417 | 2,417 | 64,236 |
|  | **qter** | 83,257,441 | 83,246,392 | 11,049 | 84,276,897 | 84,271,316 | 5,581 | 5,468 |
| **18** | **pter** | 0 | 18,868 | 18,868 | 0 | 10,647 | 10,647 | 8,221 |
|  | **qter** | 80,373,285 | 80,238,674 | 134,611 | 80,542,538 | 80,532,820 | 9,718 | 124,893 |
| **19** | **pter** | 0 | 61,294 | 61,294 | 0 | 7,043 | 7,043 | 54,251 |
|  | **qter** | 58,617,616 | 58,605,715 | 11,901 | 61,707,364 | 61,702,859 | 4,505 | 7,396 |
| **20** | **pter** | 0 | 70,156 | 70,156 | 0 | 5,239 | 5,239 | 64,917 |
|  | **qter** | 64,444,167 | 64,333,718 | 110,449 | 66,210,255 | 66,202,695 | 7,560 | 102,889 |
| **21** | **pter** | 0 | 5,010,514 | 5,010,514 ^a^ | 0 | 3,112 | 3,112 ^b^ | 5,007,402 |
|  | **qter** | 46,709,983 | 46,697,230 | 12,753 | 45,090,682 | 45,083,684 | 6,998 | 5,755 |
| **22** | **pter** | 0 | 10,514,803 | 10,514,803 ^a^ | 0 | 4,678 | 4,678 ^b^ | 10,510,125 |
|  | **qter** | 50,818,468 | 50,805,587 | 12,881 | 51,324,926 | 51,319,492 | 5,434 | 7,447 |
| **X** | **pter** | 0 | 11,554 | 11,554 | 0 | 3,343 | 3,343 | 8,211 |
|  | **qter** | 156,040,895 | 156,025,612 | 15,283 | 154,259,566 | 154,252,349 | 7,217 | 8,066 |
| **Y** | **pter** | 0 | 2,488,035 | 2,488,035 | 0 | 7,175 | 7,175 | 2,480,860 |
|  | **qter** | 57,227,415 | 26,624,345^c^ | 30,603,070 | 62,460,029 | 27,436,299^c^ | 35,023,730 | -4,420,660 |
|  |  | 57,227,415 | 57,212,132^d^ | 15,283 | 62,460,029 | 62,449,338^d^ | 10,691 | 4,592 |

Chr: chromosome; ^a^Acrocentric chromosomes short arms; ^b^The closest label to the pter region of acrocentric chromosomes may not be used for alignment due to extremely complex region; ^c^Label where most maps align contiguously; ^d^Last label of the Y chromosome.

**Supplementary Table 3. Detailed information on the ring chromosomes analyzed by optical genome mapping, including the two from the present study and the nine previously reported.**

| **Study** | **Patient** | **Ring Chromosome** | **Karyotype** | **Reference Genome** | **OGM resolution** | **Role of OGM** | **Deletion** | | **Telomeric sequences** | **LRS resolution** | **Additional information** |
| --- | --- | --- | --- | --- | --- | --- | --- | --- | --- | --- | --- |
|  |  |  |  |  |  |  | **pter** | **qter** |  |  |  |
| Present Study | 1 | 3 | 46,XY,r(3)(p26.1q29) | T2T-CHM13 | Yes | Confirmed the ring fusion and deletions | ~6 Mb | No | Yes | Yes | - |
|  | 2 | 18 | 46,XX,r(18)(p11.32q21.33) | T2T-CHM13 | Yes | Confirmed the ring fusion and deletions | No | ~18.7 Mb | No^a^ | Yes | - |
| Mantere et al. 2021 (PMID: 34237280) | 39 | X | 46,X,r(X)(p11.21q21.1)[21]/  45,X[14]/ | GRCh37/hg19 | Yes | Confirmed the ring fusion and deletions | ~57 Mb | ~77 Mb | No | No | - |
| Schuy et al. 2024 (PMID: 39669604) | RD_P26 | 21 | 46,XX,r(21)^b^ | T2T-CHM13 | Yes | Confirmed the ring fusion, detected CNVs and inv, reconstructed the p-arm | ~330 kb | ~2.4 Mb | No | Yes | - |
| Mostovoy et al. 2024^d^  (PMID: 39520989) | NA10284 | 17 | 46,XY,r(17)(p13q25) | T2T-CHM13 | Yes | Confirmed 17p-17q fusion | ~47 kb | No | Yes | Yes | - |
|  | NA06047 | 17 | 46,XY,r(17)(p13q25) | T2T-CHM13 | Yes | Confirmed both the 17p–11 fusion and the 11–17qter fusion | ~5.7 Mb | No | Yes | Yes | ~300 kb insertion of chr11 region |
|  | NA03321 | 13 | 46,XX,r(13)(p13q34) | T2T-CHM13 | Yes | Confirmed the fusion between 13q and the segmental duplication on 13p, 14p, and 21p | Yes^c^ | Yes^c^ | No | Yes | Involvement of acrocentric chromosome p arm |
|  | NA07364 | 14 | 46,XY,r(14)(p12q32.3) | T2T-CHM13 | Yes | Confirmed the 14q-rDNA fusion and captured the missing inversion breakpoint | Yes^c^ | Yes^c^ | No | Partially |  |
|  | NA21885 | 15 | 46,XY,r(15)(p12q26)[7]/  46,XY[3] | T2T-CHM13 | Yes | Confirmed the ring fusion | Yes^c^ | Yes^c^ | No | Yes |  |
|  | NA06199 | 21 | 46,XY,r(21)(p13q22) | T2T-CHM13 | Partially | Confirmed the inverted duplication breakpoint | No  (dup) | Yes^c^ | No | Partially | Dicentric ring chromosome, involvement of acrocentric chromosome p arm |
| Kim et al. 2025 (PMID: 39513527) | 1 | 17 | 46,XX,r(17)(p13q25)[16]/  46,XX[6] | GRCh37/hg19 | Yes | Identified the ring and the 17p deletion | 1.79 Mb | None | No | -^e^ | - |

OGM: optical genome mapping; LRS: long-read sequencing; PMID: PubMed Identifier; CNVs: copy number variants; inv: inversion; dup: duplication; ^a^Telomeric sequences were not present in the ring chromosome 18 according to the OGM analysis, however, the higher-resolution analysis with LRS revealed the presence of telomeric regions in the ring chromosome 18; ^b^Full karyotype information not informed; ^c^Deletion sizes not informed; ^d^Patient FQR1-P was not included in the table since OGM was not performed; ^e^LRS not performed.
